# Hypoxia regulates human mast cell adhesion to fibronectin via the PI3K/AKT signaling pathway

**DOI:** 10.1080/19336918.2020.1764690

**Published:** 2020-05-19

**Authors:** Joanna Pastwińska, Aurelia Walczak-Drzewiecka, Magdalena Łukasiak, Marcin Ratajewski, Jarosław Dastych

**Affiliations:** a Laboratory of Cellular Immunology, Institute of Medical Biology, Polish Academy of Sciences, Lodz, Poland; b Department of Experimental Immunology, Medical University of Lodz, Lodz, Poland; c Laboratory of Epigenetics, Institute of Medical Biology, Polish Academy of Sciences, Lodz, Poland

**Keywords:** Mast cells, LAD2, adhesion, fibronectin, integrin α5β1, hypoxia, SCF, PI3K, AKT, wortmannin

## Abstract

A decrease in oxygen concentration is a hallmark of inflammatory reactions resulting from infection or homeostasis disorders. Mast cells interact with extracellular matrix and other cells by adhesion receptors. We investigated the effect of hypoxia on integrin-mediated mast cell adhesion to fibronectin. We found that it was mediated by the α5/β1 receptor and that hypoxia significantly upregulated this process. Hypoxia-mediated increases in mast cell adhesion occurred without increased surface expression of integrins, suggesting regulation by inside-out integrin signaling. Hypoxia also mediated an increase in phosphorylation of Akt, and PI3’kinase inhibitors abolished hypoxia-mediated mast cell adhesion. Hypoxia upregulates the function of integrin receptors by PI3’ kinase-dependent signaling. This process might be important for the location of mast cells at inflammatory sites

## Introduction

The partial pressure of oxygen in various cell types is diverse; i.e., arterial blood, venous blood, brain, and muscle are characterized by oxygen concentrations of approximately 13.2, 5.3, 4.4, and 3.8%, respectively. All these oxygen concentrations are lower than the atmospheric concentration of 21% used as a standard in cell culture laboratories. Hypoxia is a condition in which the partial oxygen pressure drops below the physiological standard [1]. On the one hand, hypoxia itself may lead to an inflammatory process by mediating an increase in the production of pro-inflammatory cytokines, as is the case in mountain climbers exposed to low oxygen supply in breathed air or in patients experiencing ischemia. On the other hand, hypoxia may also result from ongoing inflammation, since inflamed tissues increase their oxygen consumption, creating very low oxygen concentrations locally, especially in cases of pathogen growth and development of solid tumors [2]. Changes in oxygen concentration are sensed by eukaryotic cells using specialized molecular sensors triggering signaling cascades that initiate adaptive changes in gene expression patterns. The major oxygen sensor known as hypoxia‑induced factor‑1α (HIF-1α) is a protein that is unstable under normoxic conditions but is stabilized at a lowered oxygen concentration, and following dimerization with HIF-1β, it directly regulates the expression of multiple genes, enabling cells to adjust to lower oxygen concentrations [3].

Mast cells are one of the key cell types that orchestrate the initiation and termination of inflammatory processes. They are abundant in connective tissue and mucosa and are able to produce and release a large set of inflammatory mediators, including granule-stored preformed compounds such as histamine and *de novo*-synthesized phospholipid derivatives and cytokines [4]. Mast cells express various types of receptors with affinities to a variety of ligands, including high affinity IgE receptor FcϵRI and pattern recognition receptors, such as TLR2, TLR3, TLR4, and RIG-I, which trigger mast cell activation and release of mediators [5]. Mast cells also express various adhesion molecules, including integrin receptors, that are involved in their location in tissues and their ability to infiltrate inflammatory sites [6]. Integrins are transmembrane receptors that create heterodimers consisting of one of eighteen α (α1–11, αIIb, αD, αE, αL, αM, αV, αX) and one of eight β (β1–8) subunits that are present on the cell surface either in the opened conformation of the active state or in the bent conformation of the non-active state, which results in inactivity of the receptor. The activity of certain integrins is controlled by the inside-out signaling pathway, which mediates the transition from an inactive to an active state [7]. Integrin-mediated adhesion induces various effects in mast cell physiology, including cytoskeletal reorganization, increased proliferation and differentiation, phenotype maintenance [8], and enhanced mediator secretion [8,9]. Hypoxia has been reported to upregulate surface expression of integrins in human neutrophils [10] or both functional protein and gene expression in the human myelocytic cell line U937 [11] and mouse peripheral blood mononuclear cells [12].

In this study, we investigated the effect of hypoxia on LAD2 human mast cell adhesion to fibronectin (FN). As will be shown in this paper, within minutes of exposure to hypoxic conditions, mast cells adhered to FN in increased numbers. Hypoxia-mediated mast cell adhesion was dependent on α5/β1 integrin and PI3ʹ kinase.

## Results

### LAD2 mast cells adhere to FN and express various integrin receptors

**Figure 1.**
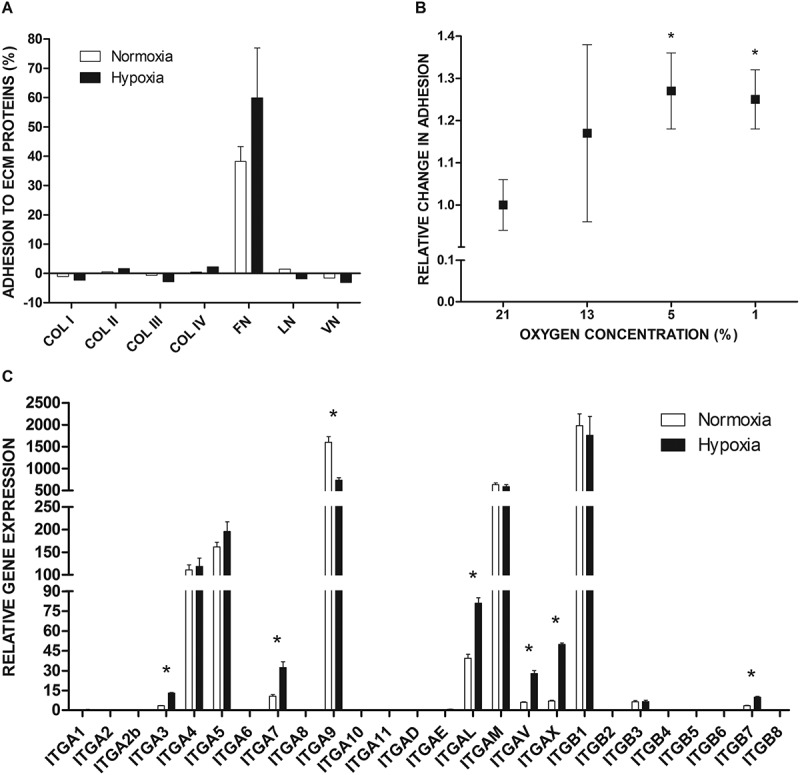
Integrin-mediated adhesion and integrin expression in LAD2 mast cells at the gene level under the influence of hypoxia. (a) LAD2 mast cell adhesion to selected extracellular matrix proteins assessed after 72 hours of incubation in 21% (Normoxia) or 5% (Hypoxia) oxygen. For FN mean ± SEM. COL I–IV, collagen type I–IV; FN, fibronectin; LN, laminin; VN, vitronectin; (b) Relative change in adhesion after incubation in different oxygen concentrations compared to atmospheric condition. Adhesion in 21% oxygen was arbitrarily accepted as 1. Mean ± SEM. * p < 0.05, paired t-test. (c) The mRNA expression of different genes encoding integrin subunits was measured after 72 hours incubation in 21% (Normoxia) or 1% (Hypoxia) oxygen. Mean ± SEM. * p < 0.05, paired t-test.

We investigated adhesion of LAD2 mast cells cultured in normal (21% oxygen) or hypoxic (5% oxygen) atmosphere to selected extracellular matrix (ECM) proteins that are ligands for integrin receptors (Figure 1(a)). Adhesion assays showed that under standard conditions, LAD2 mast cells adhered spontaneously to FN but not to collagen type I–IV, laminin, and vitronectin. Under hypoxic conditions, adhesion to FN was tended to be higher (60%) compared to the control (40%). Cells exposed to reduced oxygen concentrations, similar to the control, did not adhere to collagen type I–IV, laminin, and vitronectin (Figure 1(a)). We examined the effect of different oxygen concentrations on mast cell adhesion to FN (Figure 1(b)). Adhesion assay showed that short culture of mast cells in an atmosphere of 13% oxygen did not result in change in adhesion of these cells to FN. However, incubation in atmospheres of 1% and 5% oxygen resulted in comparable and statistically significant increase in adhesion of mast cells to FN (Figure 1(b)). Real-time PCR analysis of the expression of integrin genes showed that mast cells expressed high levels of ITGB1, ITGA5, and ITGA4 coding subunits of the classical FN receptor α5β1 and one of the alternative FN receptors α4β1 (Figure 1(c)). Culture under hypoxic conditions did not change the expression levels of any of these genes. RT-qPCR analysis of the selected genes (ITGA4, ITGA5, ITGB1) in LAD2 cells cultured in 21% or 5% oxygen for 72 h, did not show change in expression of these genes (data not shown). The expression of genes coding for subunits of other known FN receptors, such as αIIb, αV, β3, β6, β7, and β8, was undetectable or very low. Similarly, there was low or undetectable expression of genes for ITGA1, ITGA2, ITGA10, ITGA11, and ITGB2, which are necessary to form collagen receptors (α1β1, α2β1, α10β1, α11β1, αXβ2); ITGA3, ITGA6, ITGA7, and ITGB4, which are necessary to form laminin receptors (α3β1, α6β1, α6β4, α7β1); and ITGA8, ITGB3, ITGB5, and ITGB8, which are necessary to form vitronectin receptors (α8β1, αVβ3, αVβ5, αVβ8, αIIbβ3) (Figure 1(c)).

### Short-term hypoxia increases RGD-dependent adhesion to FN

**Figure 2.**
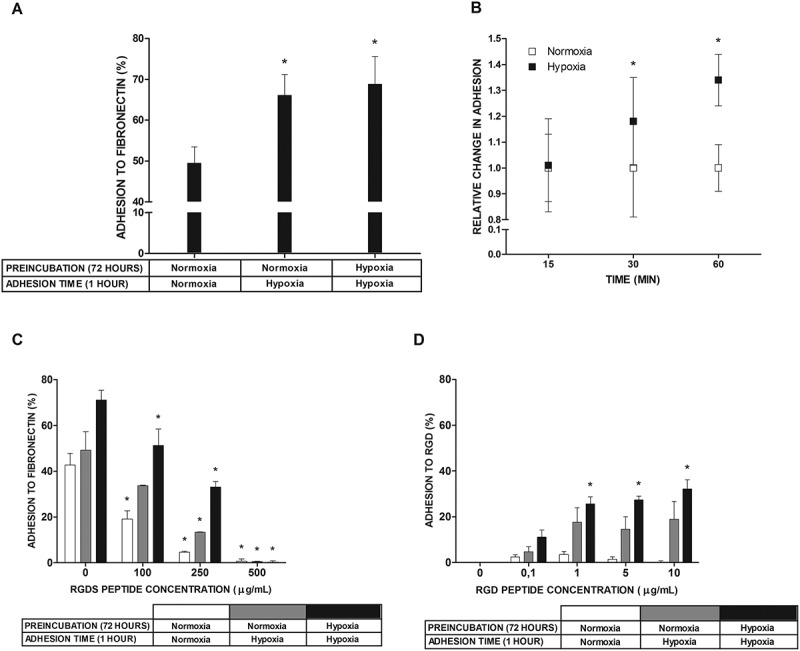
Adhesion to FN and RGD peptide under normoxic and hypoxic conditions. (a) Percentage of adhesion to FN estimated after 1 hour and 72 hour incubation in 5% (Hypoxia) oxygen. Mean ± SEM. * p < 0.05, repeated measures ANOVA followed by Tukey’s test. (b) Relative change in adhesion estimated after shorter periods of time compared with 1 hour incubation in 5% (Hypoxia) oxygen. Adhesion in 21% (Normoxia) oxygen was arbitrarily accepted as 1. Mean ± SEM. * p < 0.05, paired t-test. (c) Adhesion after 5 minutes of preincubation with different concentrations of RGDS peptide after 1 hour and 72 hours of incubation in 21% (Normoxia) and 5% (Hypoxia) oxygen. Mean ± SEM. * p < 0.05, repeated measures ANOVA followed by Dunnett’s test. (d) Percentage of adhesion to various concentrations of RGD peptide estimated after 1 hour and 72 hour incubation in 1% (Hypoxia) oxygen. Mean ± SEM. * p < 0.05, ANOVA on ranks followed by Student-Newman-Keus post hoc.

Next, we examined the effect of different culture times on mast cell adhesion to FN (Figure 2(a,b)). Resultant data showed that cells cultured in atmosphere containing 5% oxygen for 1 hour and 72 hours adhere to FN in similar numbers that are significantly higher than the number of cells adhering to FN when cultured under standard atmospheric conditions (Figure 2(a)). The kinetics of mast cell adhesion to FN following the transfer of mast cells from a standard to a hypoxic atmosphere presented in Figure 2(b) shows some statistically significant increase in mast cell adhesion already at 30 minutes. Integrin-mediated mast cell adhesion to FN [13] is based on the recognition of the RGD motif on the FN molecule. The RGDS peptide completely inhibited adhesion, regardless of the conditions (Figure 2(c)). However, the concentration of RGDS required to observe 50% inhibition was higher for adhesion observed in a hypoxic atmosphere (5% oxygen) than in a normal atmosphere (21% oxygen). We also conducted an adhesion assay using specifically engineered RGD peptide motif instead of FN. It turned out that the percentage of adhered to RGD LAD2 cells exposed to 1% oxygen, especially for 72 hours, was significantly higher than in case of cell exposed to 21% oxygen, and furthermore, the percentage of adherent cells exposed to hypoxia increased in a peptide concentration dependent manner. Cells exposed to normoxic conditions did not adhere to RGD peptide at any concentration used (Figure 2(d)).

### Mast cell adhesion to FN under hypoxic conditions depends on integrin α5β1

**Figure 3.**
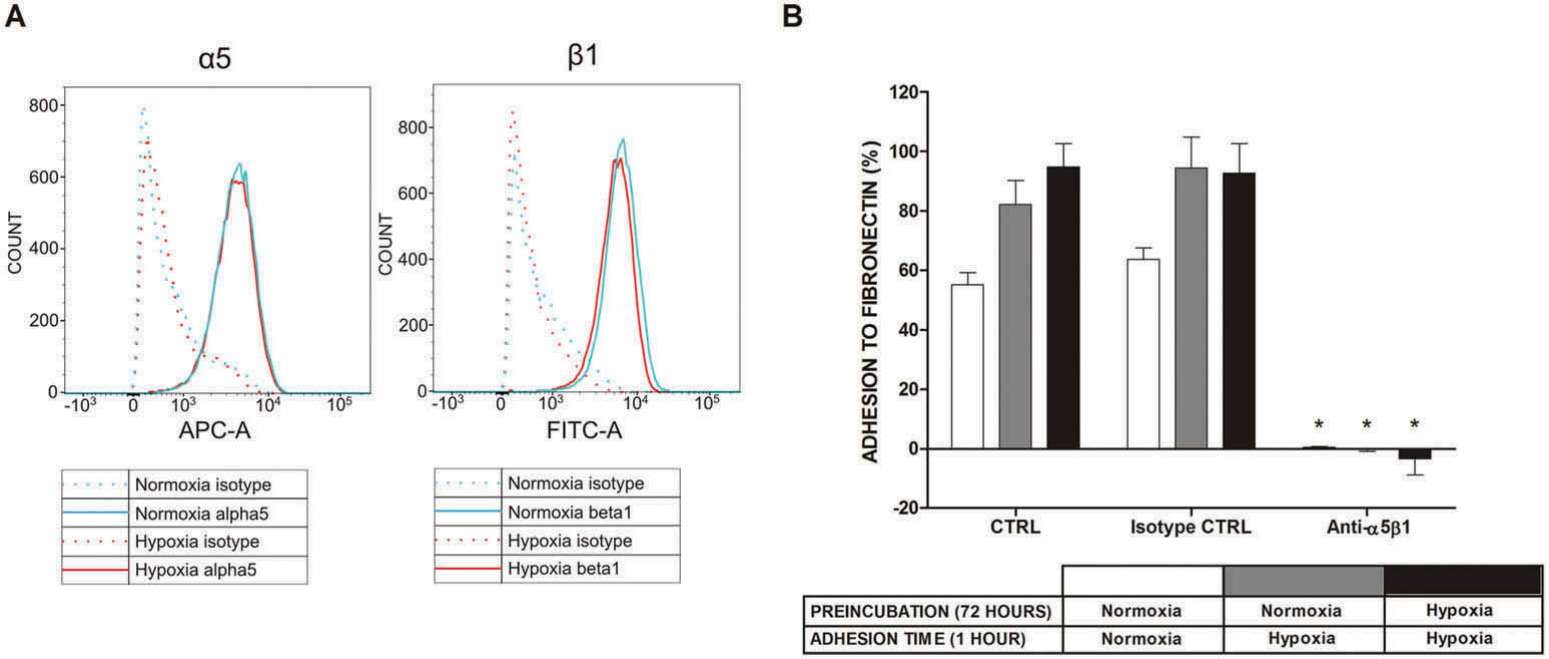
Expression of α5β1 integrin in LAD2 mast cells at the protein level and mast cell adhesion to FN after blocking this receptor under normoxic and hypoxic conditions. (a) Expression of the α5β1 integrin receptor on the surface of LAD2 mast cells was assessed under conditions of 21% and 5/1% oxygen. A representative result of 21% (Normoxia) and 1% (Hypoxia) oxygen was selected. (b) Adhesion after 30 minutes of preincubation with 10 μg/mL anti-α5β1 antibody in 21% (Normoxia) and 1% (Hypoxia) oxygen. CTRL, control (untreated cells). Mean ± SEM. * p < 0.05, paired t-test.

As seen in Figure 3(a), examination of the α5β1 receptor by flow cytometry did not show increased expression of this FN receptor on the mast cell surface following 1 hour incubation in an atmosphere containing 1% oxygen. Similar observations were made after exposing cells to 5% oxygen for 1 hour and 72 hours (data not shown). To confirm the engagement of α5β1 integrin in human mast cell adhesion to FN, we performed an adhesion assay under atmospheric and hypoxic conditions in the presence and absence of blocking antibodies. As shown in Figure 3(b), the addition of blocking antibodies at a concentration of 10 μg/mL completely inhibited the adhesion process under normoxic and hypoxic (1% O_2_) conditions. A similar level of inhibition of adhesion was observed with a lower (1 μg/mL) concentration of antibodies (data not shown).

### Wortmannin inhibited adhesion to FN under normoxic and hypoxic conditions

To investigate the signaling pathways involved in the upregulation of LAD2 mast cell adhesion by hypoxia, we screened several pharmacologic inhibitors for their effect on mast cell adhesion under hypoxic conditions. Among selected inhibitors, only wortmannin was able to significantly inhibit adhesion to FN (Table 1). While other tested inhibitors did not have a significant inhibitory effect on adhesion (though LY-294002 effects were considered as tended to be decreased), two tested compounds, namely, PF-573228 and bosutinib, upregulated mast cell adhesion (Table 1) and enhanced mast cell spreading on FN, as observed under a microscope (data not shown).

**Table 1.** LAD2 mast cell inhibition of adhesion to FN with selected inhibitors.

Inhibitor	Target protein	Conc. (μM)	Effect	Inhibition (%)	
				21% O_2_	1% O_2_
2-MeOE2*	HIF-1α	10	-	9	4
Akt 1/2 KI**	Akt1/Akt2	5	↑	0	0
AS-252,424**	PI3 Kγ	10	-	0	1
Bosutinib**	Src/Abl	1	↑↑	0	0
Cyclosporine A**	Calcineurin	10	↓	15	12
Genistein**	PTK	10	-	0	0
Go 6983**	pan-PKC	1	-	7	0
IOX2*	PHD2	50	-	0	8
LY-294,002	PI3 Kα/δ/β	5	↓	11	29
Menadione**	Cdc25 phosphatase and mitochondrial DNA polymerase γ	15	↑	1	0
Nilotinib**	Bcr-Abl	10	-	0	0
PD98059**	MEK	20	↑	0	0
PF-573,228*	FAK	5	↑↑	0	0
R406***	Syk	5	↓	13	12
SB203580**	p38 MAPK	10	↑	0	0
SP600125**	JNK	20	↑	0	0
Wortmannin**	PI3 K	1	↓↓	82	54
U0126**	MEK1/2	10	↑	0	0

KI, kinase inhibitor; -, no effect; ↓, inhibitory effect; ↓↓, strong inhibitory effect; ↑, activation effect; ↑↑, strong activation effect; *, Selleckchem; **, Sigma-Aldrich; ***, InvivoGen;

### PI3K inhibitors and deprivation of SCF significantly lowered adhesion to FN in hypoxia

**Figure 4.**
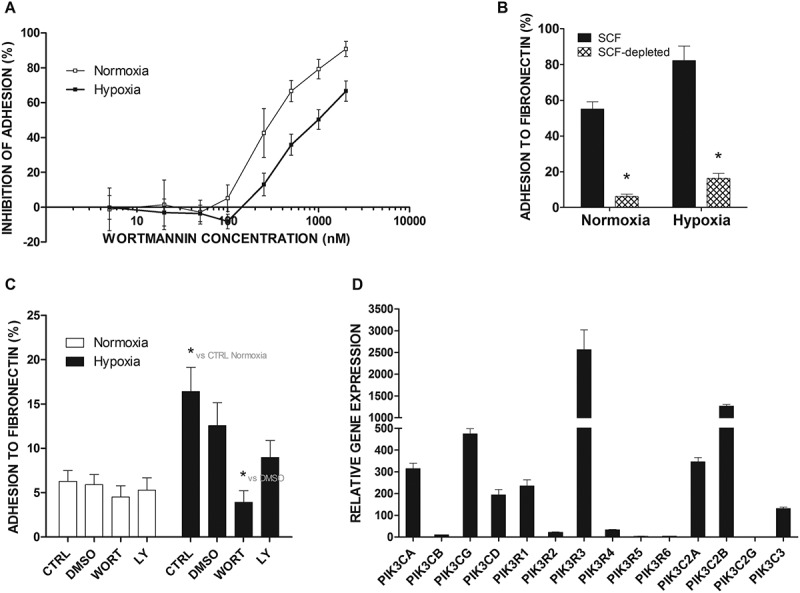
Adhesion of LAD2 and SCF-depleted LAD2 mast cells to FN after blocking the PI3 K signaling pathway and expression of the PI3 K family at the gene level. (a) Percentage of adhesion inhibition after 30 minutes of preincubation with various concentrations (5 nM, 20 nM, 50 nM, 100 nM, 250 nM, 500 nM, 1000 nM, and 2000 nM) of wortmannin in 21% (Normoxia) and 1% (Hypoxia) oxygen relative to the carrier (0.25% DMSO). The scale is presented as log10. Mean ± SEM. 21% O_2_: IC50 = 303 nM, (95% CI [141, 628]); 1% O_2_: IC50 = 919 nM, (95% CI [340, 1000]). (b) LAD2 mast cells were deprived of SCF for 72 hours. Then, adhesion was estimated after 30 minutes of preincubation with wortmannin or LY-294,002 in 21% (Normoxia) and 1% (Hypoxia) oxygen. CTRL, control (untreated cells); DMSO, cells preincubated with 0.05% DMSO; WORT, cells preincubated with 250 nM wortmannin; LY, cells preincubated with 5 μM LY-294,002; Mean ± SEM. * p < 0.05, repeated measures ANOVA followed by Dunnett’s test. (c) LAD2 mast cells were cultured in fully supplemented medium or deprived of SCF for 72 hours. Percentage of adhesion was estimated after 1 hour incubation in 21% (Normoxia) or 1% (Hypoxia) oxygen. Mean ± SEM. * p < 0.05 repeated measures ANOVA followed by Tukey’s test. (d) The mRNA expression of different PI3 K family genes was measured after 72 hours incubation in 21% oxygen. Mean ± SEM.

Based on initial observations, we further investigated the effect of different wortmannin concentrations on the adhesion of LAD2 mast cells to FN under normoxic and hypoxic (1% O_2_) conditions. As shown in Figure 4(a), wortmannin inhibited LAD2 mast cell adhesion to FN in a dose-dependent manner with IC50 = 303 nM, (95% CI [141, 628]) under normoxic conditions and IC50 = 919 nM, (95% CI [340, 1000]) under hypoxic conditions. LAD2 mast cells are SCF/c-kit-dependent [14] and were cultured in the presence of 100 ng hrSCF. The SCF/c-kit pathway is known to regulate mast cell adhesion [15,16]. Therefore, we investigated whether LAD2 mast cells deprived of SCF will also respond to hypoxia by increased adhesion to FN. Interestingly, in LAD2 mast cells cultured without SCF, hypoxia caused relatively higher upregulation of adhesion to FN compared to standard culture conditions (2.7-fold compared to 1.4-fold increase, Figure 4(b)). As shown in Figure 4(c), LAD2 mast cells depleted for 72 hours before the adhesion test adhered spontaneously to FN in significantly lower numbers than those cultured in the presence of SCF (Figure 4(b)). Similar to cells cultured in standard media, in cells cultured without SCF, wortmannin, at a concentration of 250 nM, completely inhibited the upregulation of mast cell adhesion to FN under hypoxic conditions. Under such conditions, another PI3 K inhibitor, LY-294002 (5 μM), also inhibited adhesion to FN under hypoxia but to a lower extent than wortmannin (Figure 4(b)). As seen in Figure 4(d), gene expression analysis under normoxic conditions showed that LAD2 mast cells express most of the PI3K genes, specifically the class I, catalytic and regulatory groups, which are responsible for signaling of the PI3K/AKT/mTOR pathway (Figure 4(d)).

### PI3K/AKT signaling pathway participates in increased adhesion to FN in hypoxia

**Figure 5.**
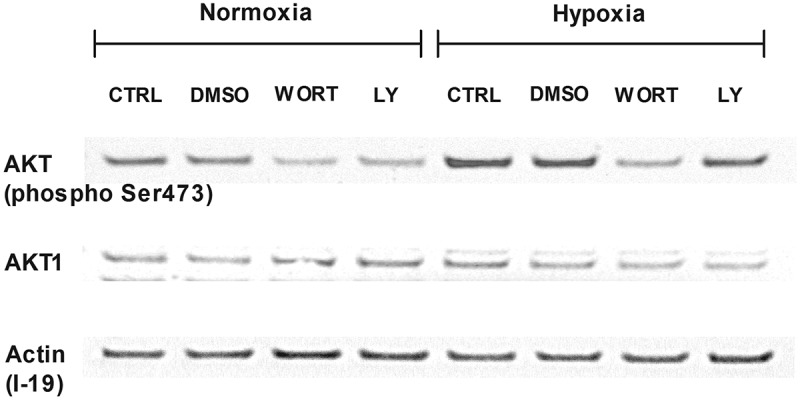
Involvement of the PI3 K/AKT signaling pathway in hypoxia. Western blot analysis of phosphorylated AKT and whole AKT1 and their expression in LAD2 mast cells. Cells were deprived of SCF for 72 hours. Then, protein isolates were collected after 30 minutes of preincubation with wortmannin (250 nM) or LY-294,002 (5 μM) and 1 hour in 21% (Normoxia) and 1% (Hypoxia) oxygen. CTRL, control (untreated cells); DMSO, cells preincubated with 0.05% DMSO; WORT, cells preincubated with 250 nM wortmannin; LY, cells preincubated with 5 μM LY-294,002.

We decided to determine whether hypoxia resulted in activation of the PI3 K/AKT pathway in LAD2 mast cells. To this end, we determined the phosphorylation of AKT protein in LAD2 mast cells incubated under normoxic and hypoxic (1% O_2_) conditions in media depleted of SCF and in the absence and presence of PI3K inhibitors. As shown in Figure 5, hypoxia resulted in an increased signal for phosphorylated AKT compared to the control, with a comparable signal for total AKT protein. The presence of PI3K inhibitors (wortmannin 250 nM and LY-294002 5 μM) inhibited the increase in signal for phosphorylated AKT in LAD2 mast cells exposed to hypoxia (Figure 5).

## Discussion

We observed human LAD2 mast cell adhesion to FN (Figure 1(a)) in a process mediated by α5β1 integrin and the RGD motif on FN (Figure 2(c,d), Figure 3(b)). This is consistent with the expression pattern of integrin genes, where ITGA5 and ITGB1 exhibited high levels of expression, while the expression of genes necessary for assembling other functional FN binding receptors, such as α4β7, αVβ1, αVβ3, αVβ6, αVβ8, and αIIbβ3, was negligible (Figure 1(c)). Mast cell adhesion to FN mediated by the classical α5β1 integrin receptor recognizing the RGD motif on FN has been previously observed in different experimental models [8], including mouse [13], rat [17], and human mast cells [18]. Integrin α4β1 is a receptor that recognizes fibronectin. We found that its expression in LAD2 cells is slightly lower than α5β1 (Figure 1(c)). Nevertheless, unlike α5β1 that recognizes the RGD motif in fibronectin, integrin α4β1 binds to the connecting segment 1 (CS-1) domain [19,20] of the fibronectin. We also examined the effect of CS-1 peptide on mast cell adhesion to fibronectin and we did not observe a significant decrease in adhesion (data not shown), as was in the case of RGDS peptide usage. Interestingly, there is a very high expression of genes encoding integrins α9 and αM. However, none of these subunits in combination with the appropriate beta subunit recognize fibronectin as a ligand [21,22]. The α9β1 receptor binds to tenascin and osteopontin, while αMβ2 recognizes fibrinogen [21,22]. Furthermore, ITGB2 expression in LAD2 cells is negligible, and thus, we suppose that it is not possible to form a fully functional αMβ2 receptor. Unlike other types of human mast cells that have been reported to adhere to collagen type I, III, and IV, laminin, and vitronectin [18,23–26], LAD2 mast cells did not adhere to these ECM proteins (Figure 1(a)). The lack of interaction of human LAD2 mast cells with these adhesion ligands is consistent with the observed expression pattern of integrin genes, showing negligible expression of at least one of the genes for each pair that codes for integrin subunits necessary to assemble receptors for collagen (α1β1, α2β1, α10β1, α11β1, αXβ2), laminin (α1β1, α2β1, α3β1, α6β1, α6β4, α7β1, α10β1), and vitronectin (α8β1, αVβ3, αVβ5, αVβ8, αIIbβ3) (Figure 1(c)).

LAD2 mast cells are adapted to growth under atmospheric oxygen concentration [14]. We observed that short exposure of LAD2 mast cells to hypoxic conditions (1% to 5% oxygen) upregulated mast cell adhesion to FN (Figure 1(b), Figure 2(a,b)). This upregulation of adhesion occurred without increased expression of α5β1 integrin receptors on the mast cell surface (Figure 3(a)). At the same time, mast cell adhesion under hypoxic conditions was abolished by anti-α5β1 mAb (Figure 3(b)). This suggests that hypoxia upregulated human mast cell adhesion to FN by changing the affinity of α5β1 integrins, rather than increasing their number on the mast cell surface. Similar regulation of mast cell adhesion to FN by inside-out integrin signaling was previously observed for other activators, such as antigen [27], IgE [28], aggregated IgG [29], cytokines [15,30], and biomaterials [31].

Analysis of the sensitivity of hypoxia-mediated mast cell adhesion to FN to selected pharmacologic inhibitors showed that this adhesion process is inhibited by the PI3ʹ kinase inhibitor wortmannin in a dose-dependent manner (Figure 4(a)), and real-time RT-PCR analysis showed that LAD2 mast cells expressed several catalytic subunits of PI3ʹ kinase that are known to be inhibited by wortmannin (Figure 4(d)). These observations suggest that hypoxia upregulates mast cell adhesion by activating the signaling process that involves PI3ʹ kinase. One potential pathway known to regulate integrin-mediated mast cell adhesion in a PI3ʹ kinase-dependent fashion is SCF/c-Kit signaling, which is also critical for the survival and proliferation of human mast cells, including the LAD2 cell line. Interestingly, SCF/c-Kit signaling regulation of cell survival, migration, and adhesion involves PI3ʹ kinase [15,32–36]. For that reason, one potential hypothesis linking PI3ʹ kinase activity with hypoxia-mediated upregulation of mast cell adhesion is enhancement of SCF/c-Kit signaling. As shown in Figure 4(b,c), depletion of SCF from human mast cells for 72 hours resulted in significantly lower adhesion compared to cells cultured in the presence of SCF. At the same time, the transfer of SCF-depleted mast cells to hypoxic conditions resulted in the upregulation of cell adhesion to FN. Thus, while spontaneous adhesion of LAD2 mast cells under normoxic conditions was dependendent on the presence of SCF, hypoxia mediates adhesion to FN independently of SCF/c-Kit signaling. Furthermore, under such conditions, short exposure of mast cells to hypoxia mediated increased phosphorylation of Akt (Figure 5), a downstream protein in the PI3ʹ kinase signaling pathway, and this upregulation of protein phosphorylation was abolished by PI3ʹ kinase inhibitors. Taken together, these observations suggest the regulation of the mast cell adhesion process by a PI3ʹ kinase-dependent signaling pathway other than the SCF/c-Kit pathway. PI3ʹ kinase/Akt signaling has been previously implicated in signaling processes induced by hypoxia in different cells, such as skeletal muscle [37] and glioma [38].

In summary, we report here the novel mode of regulation of mast cell adhesion to FN by a hypoxia-induced signaling process involving PI3ʹ kinase and α5/β1 integrin receptors. This regulation might be an important cellular mechanism determining mast cell distribution in tissues. There are multiple observations of an increased number of mast cells in certain diseased tissues, such as airways of patients with asthma and COPD [39–42], nasal polyps [43], inflamed periodontal area [44], and stroma of solid tumors [45], possibly due to increased mast cell migration [43,46]. Upregulation of integrin-mediated mast cell adhesion by hypoxia could provide a mechanism for retaining mast cells in areas of inflammatory reaction.

## Materials and methods

### Cell line and cell culture

The human mast cell line LAD2 was a gift from Dr. A. S. Kirshenbaum (National Institutes of Health, Bethesda, MD) [14]. Cells were cultured at 37°C in 5% CO_2_ and passaged weekly in StemPro-34 serum-free medium (Gibco) supplemented with StemPro-34 Nutrient Supplement (Gibco), 2 mM L-glutamine (Sigma-Aldrich), 100 U/mL penicillin/100 μg/mL streptomycin (Gibco), and 100 ng/mL recombinant human SCF (R&D Systems). A density of 500 000 cells/1 mL was maintained for each passage.

### Adhesion assay

Human LAD2 mast cells were cultured for 72 hours in normoxic (21% O_2_) or hypoxic (1%, 5% or 13% O_2_) conditions in medium supplemented with 100 ng/mL SCF or without this growth factor. Exposure of LAD2 cells to 1% and 5% oxygen gave similar results in comparison to the results obtained in 21% oxygen, conditions 1% and 5% were used interchangeably as hypoxia. Cells were washed three times and resuspended in StemPro-34 containing 0.1% BSA. LAD2 mast cells were seeded and cultured for 1 hour in normoxic or hypoxic conditions at a density of 50 000 cells/100 μL/well in separate 96-well plates (Nunc, Thermo Fisher Scientific). Plates were precoated with 25 µg/mL FN (Sigma-Aldrich), laminin, vitronectin, collagen type I–IV (Merck Millipore) or 0.1/1/5/10 µg/mL RGD peptide (Cell Guidance Systems), washed three times with HBSS, blocked for 3 hours at 37°C with 5% BSA and washed three times with StemPro/0.1% BSA before the adhesion assay. For studying short-term hypoxia, some of the normoxic cells were taken to hypoxia for only 1 hour of adhesion. To determine the percentage of adherent cells, we modified the Acid Phosphatase Assay Kit (Abcam, ab83367) protocol. The principle of the test is to measure the activity of acid phosphatase, which is an enzyme present in the lysosomes of most human cells [47]. This activity is evaluated by measuring OD 405 nm of a yellow product, para-nitrophenol (pNP), resulting from the hydrolysis of para-nitrophenylphosphate (pNPP). The modifications were made to adapt the method to perform the entire test on a 96-well plate, bypassing the preparation of individual samples in the tubes. Adherent cells were directly lysed on the plate, and to improve the process we supplemented assay buffer with 0.1% Triton X-100. Each sample contained 50 000 cells and the results are presented as a percentage of adhering cells. We washed away non-adherent cells three times with StemPro-34/0.1% BSA and performed all remaining steps in normoxic conditions. Adherent cells were lysed with 80 μL of assay buffer supplemented with 0.1% Triton X-100 per well. Reference samples were initially prepared in a U-bottom 96-well plate and then transferred to wells in test plates. Next, we added 50 μL of 5 mM pNPP dissolved in assay buffer/0.1% Triton X-100 per well. The plates were incubated for 1 hour at 25°C in the dark. The reaction was stopped by adding 20 μL of stop solution per well, and the OD 405 nm was measured. The percentage of adherent cells was calculated on the basis of total cell lysates. For studying the effects of 100/250/500 µg/mL RGDS peptide (Tocris Bioscience), 10 μg/mL anti-α5β1 integrin (Novus Biologicals) and inhibitors, cells were pretreated for 5 (peptide) and 30 (antibody/inhibitors) minutes before performing the adhesion assay. The list of inhibitors and final concentrations used are presented in Table 1 and Figure 4 in the Results section. Inhibitor concentrations were selected on the basis of literature data. For wortmannin and LY-294002, concentrations 250 nM and 5 µM, respectively, were selected from a series of 8 dilutions.

### FACs

Cells were fixed in normoxic (21% O_2_) or hypoxic (1% or 5% O_2_) conditions for 5 minutes in 500 μL of flow cytometry fixation buffer (R&D Systems) and washed in stain buffer containing BSA (BD Pharmingen). Cells were resuspended in 100 μL of stain buffer and incubated for 45 minutes without antibodies or with nonspecific fluorescence labeled antibody (Mouse IgG1 κ Isotype Control-Alexa Fluor 647, BD Pharmingen) at 1:20 dilution, with fluorescence labeled antibody against integrin α5 (Mouse Anti-Human CD49e-Alexa Fluor 647, BD Pharmingen) at 1:20 dilution, with primary nonspecific antibody (Purified Rat IgG2a κ Isotype Control, BD Pharmingen) at 1:25 dilution or with primary antibody against integrin β1 (Purified Rat Anti-Human CD29, BD Pharmingen) at 1:25 dilution followed by incubation for 45 minutes with fluorescence labeled secondary antibody (Goat Anti-Rat Ig-FITC, BD Pharmingen) at 1:50 dilution. Cells were washed with stain buffer and resuspended in 1 mL of stain buffer. Flow cytometry was performed using the BD LSRFortessa^TM^ (BD Biosciences).

### RT-qPCR

LAD2 mast cells were cultured for 72 hours in normoxic (21% O_2_) or hypoxic (1% O_2_) conditions. Total RNA was isolated using TRI Reagent (Sigma-Aldrich) according to the manufacturer’s protocol. Next, 5 μg of total RNA was converted to cDNA with the Maxima First Strand cDNA Synthesis Kit for RT-qPCR (Thermo Fisher Scientific) according to the manufacturer’s protocol. The expression of genes encoding all integrins in samples from normoxia and hypoxia was measured using the LightCycler 480 Probes Master mix kit (Roche) in a 384-well plate according to the manufacturer’s protocol. Reaction conditions were as follows: initial denaturation at 95°C for 5 minutes; 45 cycles of 95°C for 10 seconds, 55°C for 10 seconds, and 72°C for 20 seconds; melting curve at 95°C for 30 seconds, 72°C for 45 seconds, and 97°C; and cooling at 40°C for 15 seconds. Three housekeeping genes (HPRT1, HMBS, RPL13A) were used as internal controls. Data were analyzed and shown as relative gene expression using the ΔΔct method. HPRT1, HMBS, RPL13A primer sequences were taken from the work of Vandesompele et al. [48]. Integrin and PI3K primer sequences were published elsewhere: ITGA1, ITGA5, ITGA7, ITGA8, ITGA9, ITGA11, ITGAV, ITGB1, ITGB3, ITGB4 [49], ITGA2 [50], ITGA3 [51], ITGA4 [52], ITGAE [53], ITGB2 [54], ITGB5 [55], PIK3CA [56], PIK3CB [57], PIK3CD, PIK3R3 [58], PIK3R1 [59], PIK3R4 [60], PIK3C2B [61]. Other sequences were design using Primer3 software [62] or PrimerBlast [63]. Primer sequences are presented in Table 2.

**Table 2.** Human-specific primer sequences.

Gene	Forward primer	Reverse primer	Amplicon	Ref.
HPRT	5ʹ-tgacactggcaaaacaatgca-3’	5ʹ-ggtccttttcaccagcaagct-3’	94	[48]
HMBS	5ʹ-ggcaatgcggctgcaa-3’	5ʹ-gggtacccacgcgaatcac-3’	64	[48]
RPL13A	5ʹ-cctggaggagaagaggaaagaga-3’	5ʹ-ttgaggacctctgtgtatttgtcaa-3’	126	[48]
ITGA1	5ʹ-ggccgtagttaaagtgacc-3’	5ʹ-gtgaatctagggtgacacg-3’	185	[49]
ITGA2	5ʹ-gggcattgaaaacactcgat-3’	5ʹ-tcggatcccaagattttctg-3’	183	[50]
ITGA2b	5ʹ-ggaagctcaggtgtggac-3’	5ʹ-catcatcttcttccaggggt-3’	173	
ITGA3	5ʹ-ccgagtcaatgtccacag-3’	5ʹ-gctgggctaccctattcc-3’	87	[51]
ITGA4	5ʹ-ttccagagccaaatccaagagtaa-3’	5ʹ-aagccagccttccacataacat-3’	184	[52]
ITGA5	5ʹ-gagcagaaccatgtgtacc-3’	5ʹ-caaagtagtcacagctcagg-3’	181	[49]
ITGA6	5ʹ-cccagatattgcagttggag-3’	5ʹ-gaatctgagagggaaccaac-3’	198	
ITGA7	5ʹ-gactcactgcactactcagg-3’	5ʹ-cagctctacctccagttcc-3’	192	[49]
ITGA8	5ʹ-gagattcggtagtgctatgg-3’	5ʹ-actccttgcagaacttgg-3’	168	[49]
ITGA9	5ʹ-actggagaggaggagagg-3’	5ʹ-ccccaaagctagatacagg-3’	195	[49]
ITGA10	5ʹ-gtgagagcagcaaagaacc-3’	5ʹ-cctctccatcatgggactc-3’	166	
ITGA11	5ʹ-catcctgaagacacctaagc-3’	5ʹ-ctggcattgatctgaacc-3’	183	[49]
ITGAD	5ʹ-gctgcttttgccagattg-3’	5ʹ-cttcacagaggccatcttg-3’	188	
ITGAE	5ʹ-ggaacttctatgaaaagtgttttgag-3’	5ʹ-ctgtcccgaaggtcaaactc-3’	88	[53]
ITGAL	5ʹ-ggaaggaccctgatgctc-3’	5ʹ-gcggatgatgtctttggc-3’	203	
ITGAM	5ʹ-gtgaagccaataacgcagc −3’	5ʹ-tctccatccgtgatgacaac −3’	137	
ITGAV	5ʹ-cagtcccatctcaaatcc-3’	5ʹ-ctggccctgtataagatagc-3’	160	[49]
ITGAX	5ʹ-ggtttggagacagcgtgg-3’	5ʹ-gacatgttcacggcctcc-3’	166	
ITGB1	5ʹ-cggacagtgtgtttgtagg-3’	5ʹ-cagtgtagttggggttgc-3’	161	[49]
ITGB2	5ʹ-caacatccagcccatcttc-3’	5ʹ-gaccacattgctggagtc-3’	118	[54]
ITGB3	5ʹ-tacaaacacgtgctgacg-3’	5ʹ-gagtcttggcatcagtgg-3’	196	[49]
ITGB4	5ʹ-ctgcacctacagctacac-3’	5ʹ-cacagtacttccagcatagc-3’	173	[49]
ITGB5	5ʹ-agccagagtgtggaaacacc-3’	5ʹ-caagcagcttccagatagcc-3’	105	[55]
ITGB6	5ʹ-caagctgctgtgtgtaagg-3’	5ʹ-gacagagcccgtcattagg-3’	139	
ITGB7	5ʹ-cagcacagagtttgactaccc-3’	5ʹ-gcactggtgacagcaaag-3’	90	
ITGB8	5ʹ-ccctcacaatttgtctcagg-3’	5ʹ-caagtagcttgggctgga-3’	115	
PIK3CA	5ʹ-gctaaagaggaacactgtcc-3’	5ʹ-ggtactggccaaagattca-3’	104	[56]
PIK3CB	5ʹ-catcactctctttgcgct-3’	5ʹ-cctgagcgcctcatcaa-3’	151	[57]
PIK3CG	5ʹ-gtagaggcaaacatccagc-3’	5ʹ-tactgaactcaagccacac-3’	106	
PIK3 CD	5ʹ-ccaacctcagcaccatca-3’	5ʹ-ccgctgtctggttgatg-3’	114	[58]
PIK3R1	5ʹ-tttgccgagccctataact-3’	5ʹ-tgcatatactgggtaggctag-3’	120	[59]
PIK3R2	5ʹ-cgaggaacgcacttggt-3’	5ʹ-tccactaccacggagca-3’	135	
PIK3R3	5ʹ-ggagattatactttgactttgcg-3’	5ʹ-ggtgatagtggttaatgagct-3’	133	[58]
PIK3R4	5ʹ-cagacatcttcacatgcgtc-3’	5ʹ-tgtcattccctgtgagagc-3’	115	[60]
PIK3R5	5ʹ-cgctacgtgttgtggtct-3’	5ʹ-attgttctccagccgcc-3’	86	
PIK3R6	5ʹ-gcactatttccacgccg-3’	5ʹ-gcagtaaatctcctccagc-3’	100	
PIK3C2A	5ʹ-actcattgcttcaccagtg-3’	5ʹ-ctcaatccaggtcacagcta-3’	110	
PIK3C2B	5ʹ-ctccctaattccaaggatcag-3’	5ʹ-tcaatggagcttctttctcc-3’	74	[61]
PIK3C2 G	5ʹ-cgaccactgtttgggga-3’	5ʹ-ctgtggtcctcttcatgct-3’	120	
PIK3C3	5ʹ-ggctgcacaacagacatt-3’	5ʹ-gggataagttccacatctgac-3’	150	

### Western blot analysis

Cells were cultured for 72 hours without SCF and pretreated with 0.05% DMSO, 250 nM wortmannin or 5 μM LY-294002 for 30 minutes, and incubated for 1 hour in normoxic (21% O_2_) or hypoxic (1% O_2_) conditions. Next, cells were washed in ice-cold PBS and lysed with ice-cold RIPA buffer with 2 X cOmplete™ Mini EDTA-free Protease Inhibitor Cocktail (Roche) for 30 minutes. Protein concentrations were estimated using the Pierce™ BCA Protein Assay Kit (Thermo Fisher Scientific) according to the manufacturer’s protocol. Equal amounts of protein (25 μg) were analyzed on NuPAGE™ 4–12% Bis-Tris Protein Gels (1.5 mm, 10-well) (Thermo Fisher Scientific) followed by protein transfer to nitrocellulose membranes according to the NuPAGE® Bis-Tris Gel Instruction Booklet (Invitrogen). Membranes were blocked in Tris-buffered saline containing 0.1% Tween 20 (TBST) and 5% skimmed milk for 1 hour at room temperature. Next, membranes were immunoblotted with the primary antibodies AKT1 and AKT phospho Ser473 (GeneTex) at a 1:1000 dilution overnight at 4°C. Blots were washed and incubated with the horseradish peroxidase-conjugated secondary antibody goat anti-rabbit IgG (Abcam) at a 1:5000 dilution for 1 hour at room temperature. Antibodies were diluted in TBST/5% skimmed milk. Next, the membranes were washed and incubated with SuperSignal™ West Pico PLUS Chemiluminescent Substrate (Thermo Fisher Scientific) for 5 minutes in the dark. Reactive protein signals were visualized with the G-BOX Chemi IR6 system and GeneSys software (Syngene). As a reference, the blots were stripped and immunoblotted with β-actin antibody at a dilution of 1:100.

### Statistical analysis

All data are presented as the mean ± SEM. Statistica (version 13.1) was used to perform statistical analysis. A paired t-test or repeated measures ANOVA followed by Dunnett’s or Tukey’s post hoc test was used to detect statistically significant differences. Significant differences are declared at P < 0.05. IC50 was determined using nonlinear regression with variable slope (four parameters) in GraphPad Prism (version 8.1.2).

## References

[1] Carreau A, El Hafny-Rahbi B, Matejuk A, et al. Why is the partial oxygen pressure of human tissues a crucial parameter? Small molecules and hypoxia. J Cell Mol Med. 2011;15(6):1239–1253. 21251211 10.1111/j.1582-4934.2011.01258.x PMC4373326

[2] Eltzschig HK, Carmeliet P. Hypoxia and inflammation. N Engl J Med. 2011;364(7):656–665. 21323543 10.1056/NEJMra0910283 PMC3930928

[3] Zhang Z, Yao L, Yang J, et al. PI3K/AKT and HIF‑1 signaling pathway in hypoxia‑ischemia (Review). Mol Med Rep. 2018;18(4):3547–3554. 30106145 10.3892/mmr.2018.9375 PMC6131612

[4] Pastwińska J, Agier J, Dastych J, et al. Mast cells as the strength of the inflammatory process. Pol J Pathol. 2017;68(3):187–196. 29363910 10.5114/pjp.2017.71526

[5] da Silva EZM, Jamur MC, Oliver C. Mast cell function: a new vision of an old cell. J Histochem Cytochem. 2014;62(10):698–738. 25062998 10.1369/0022155414545334 PMC4230976

[6] Misiak-Tłoczek A, Brzezińska-Błaszczyk E. Regulacja migracji komórek tucznych. Część 1: cząsteczki adhezji międzykomórkowej. Postepy Hig Med Dosw. 2007;61:485–492. 17909516

[7] Hynes RO, Hughes H. Integrins: bidirectional, allosteric signaling machines. Cell. 2002;110(6):673–687. 12297042 10.1016/s0092-8674(02)00971-6

[8] Hamawy MM, Mergenhagen SE, Siraganian RP. Adhesion molecules as regulators of mast-cell and basophil function. Immunol Today. 1994;15(2):62–66. 8155264 10.1016/0167-5699(94)90135-X

[9] Krüger-Krasagakes S, Grützkau A, Krasagakis K, et al. Adhesion of human mast cells to extracellular matrix provides a co-stimulatory signal for cytokine production. Immunology. 1999;98(2):253–257. 10540224 10.1046/j.1365-2567.1999.00865.x PMC2326919

[10] Khawaja AA, Pericleous C, Ripoll VM, et al. Hypoxia modulates neutrophil integrin expression, adhesion and trans-endothelial migration. Poster session presented at: 2016 ACR/ARHP Annual Meeting; 2016 11 11–16;Washington, DC.

[11] Kong T, Eltzschig HK, Karhausen J, et al. Leukocyte adhesion during hypoxia is mediated by HIF-1-dependent induction of β2 integrin gene expression. Proc Natl Acad Sci U S A. 2004;101(28):10440–10445. 15235127 10.1073/pnas.0401339101 PMC478589

[12] Kubo M, Li T-S, Kamota T, et al. Increased expression of CXCR4 and integrin αM in hypoxia-preconditioned cells contributes to improved cell retention and angiogenic potency. J Cell Physiol. 2009;220(2):508–514. 19415696 10.1002/jcp.21803

[13] Dastych J, Costa JJ, Thompson HL, et al. Mast cell adhesion to fibronectin. Immunology. 1991;73(4):478–484. 1916899 PMC1384580

[14] Kirshenbaum AS, Akin C, Wu Y, et al. Characterization of novel stem cell factor responsive human mast cell lines LAD 1 and 2 established from a patient with mast cell sarcoma/leukemia; activation following aggregation of FcϵRI or FcγRI. Leuk Res. 2003;27(8):677–682. 12801524 10.1016/s0145-2126(02)00343-0

[15] Dastych J, Metcalfe DD. Stem cell factor induces mast cell adhesion to fibronectin. J Immunol. 1994;152(1):213–219. 7504710

[16] Lorentz A, Schuppan D, Gebert A, et al. Regulatory effects of stem cell factor and interleukin-4 on adhesion of human mast cells to extracellular matrix proteins. Blood. 2002;99(3):966–972. 11807000 10.1182/blood.v99.3.966

[17] Yasuda M, Hasunuma Y, Adachi H, et al. Expression and function of fibronectin binding integrins on rat mast cells. Int Immunol. 1995;7(2):251–258. 7734420 10.1093/intimm/7.2.251

[18] Columbo M, Bochner BS, Marone G. Human skin mast cells express functional beta 1 integrins that mediate adhesion to extracellular matrix proteins. J Immunol. 1995;154(11):6058–6064. 7538541

[19] White ES, Thannickal VJ, Carskadon SL, et al. Integrin α4β1 regulates migration across basement membranes by lung fibroblasts. Am J Respir Crit Care Med. 2003;168(4):436–442. 12791582 10.1164/rccm.200301-041OC PMC1997294

[20] Wu L, Bernard-Trifilo JA, Lim Y, et al. Distinct FAK-Src activation events promote alpha5beta1 and alpha4beta1 integrin-stimulated neuroblastoma cell motility. Oncogene. 2008;27(10):1439–1448. 17828307 10.1038/sj.onc.1210770 PMC2593630

[21] Hynes RO, Naba A. Overview of the matrisome–an inventory of extracellular matrix constituents and functions. Cold Spring Harb Perspect Biol. 2012;4(1):a004903. 21937732 10.1101/cshperspect.a004903 PMC3249625

[22] Siebers MC, Walboomers XF, van den Dolder J, et al. The behavior of osteoblast-like cells on various substrates with functional blocking of integrin-beta1 and integrin-beta3. J Mater Sci Mater Med. 2008;19(2):861–868. 17665129 10.1007/s10856-007-0166-6 PMC2233710

[23] Trautmann A, Feuerstein B, Ernst N, et al. Heterotypic cell–cell adhesion of human mast cells to fibroblasts. Arch Dermatol Res. 1997;289(4):194–203. 9143735 10.1007/s004030050180

[24] Kruger-Krasagakes S, Grutzkau A, Baghramian R, et al. Interactions of immature human mast cells with extracellular matrix: expression of specific adhesion receptors and their role in cell binding to matrix proteins. J Invest Dermatol. 1996;106(3):538–543. 8648190 10.1111/1523-1747.ep12343953

[25] Tachimoto H, Hudson SA, Bochner BS. Acquisition and alteration of adhesion molecules during cultured human mast cell differentiation. J Allergy Clin Immunol. 2001;107(2):302–309. 11174197 10.1067/mai.2001.111930

[26] Shimizu Y, Irani A-MA, Brown EJ, et al. Human mast cells derived from fetal liver cells cultured with stem cell factor express a functional CD5l/CD61 (αVβ3) integrin. Blood. 1995;86(3):930–939. 7542504

[27] Dastych J, Wyczółkowska J, Metcalfe DD. Characterization of α5-integrin-dependent mast cell adhesion following FcϵRI aggregation. Int Arch Allergy Immunol. 2001;125(2):152–159. 11435732 10.1159/000053809

[28] Lam V, Kalesnikoff J, Lee CWK, et al. IgE alone stimulates mast cell adhesion to fibronectin via pathways similar to those used by IgE + antigen but distinct from those used by steel factor. Blood. 2003;102(4):1405–1413. 12702510 10.1182/blood-2002-10-3176

[29] Dastych J, Hardison MC, Metcalfe DD. Aggregation of low affinity IgG receptors induces mast cell adherence to fibronectin: requirement for the common FcR gamma-chain. J Immunol. 1997;158(4):1803–1809. 9029119

[30] Lévesque J-P, Leavesley DI, Niutta S, et al. Cytokines increase human hemopoietic cell adhesiveness by activation of very late antigen (VLA)-4 and VLA-5 integrins. J Exp Med. 1995;181(5):1805–1815. 7536795 10.1084/jem.181.5.1805 PMC2192007

[31] Lüthen F, Lange R, Becker P, et al. The influence of surface roughness of titanium on β1- and β3-integrin adhesion and the organization of fibronectin in human osteoblastic cells. Biomaterials. 2005;26(15):2423–2440. 15585246 10.1016/j.biomaterials.2004.07.054

[32] Serve H, Yee NS, Stella G, et al. Differential roles of PI3-kinase and Kit tyrosine 821 in Kit receptor-mediated proliferation, survival and cell adhesion in mast cells. Embo J. 1995;14(3):473–483. 7532131 10.1002/j.1460-2075.1995.tb07023.x PMC398105

[33] Blume-Jensen P, Janknecht R, Hunter T. The kit receptor promotes cell survival via activation of PI 3-kinase and subsequent Akt-mediated phosphorylation of Bad on Ser136. Curr Biol. 1998;8(13):779–782. 9651683 10.1016/s0960-9822(98)70302-1

[34] Vajravelu BN, Hong KU, Al-Maqtari T, et al. C-Kit promotes growth and migration of human cardiac progenitor cells via the PI3K–AKT and MEK–ERK pathways. PLoS One. 2015;10(10):e0140798. 26474484 10.1371/journal.pone.0140798 PMC4608800

[35] Guo J, Jie W, Shen Z, et al. SCF increases cardiac stem cell migration through PI3K/AKT and MMP‑2/‑9 signaling. Int J Mol Med. 2014;34(1):112–118. 24804928 10.3892/ijmm.2014.1773 PMC4072340

[36] Dastych J, Taub D, Hardison MC, et al. Tyrosine kinase-deficient W^v^ c-kit induces mast cell adhesion and chemotaxis. Am J Physiol. 1998;275(5):C1291–1299. 9814978 10.1152/ajpcell.1998.275.5.C1291

[37] Gan Z, Powell FL, Zambon AC, et al. Transcriptomic analysis identifies a role of PI3K–Akt signalling in the responses of skeletal muscle to acute hypoxia in vivo. J Physiol. 2017;595(17):5797–5813. 28688178 10.1113/JP274556 PMC5577531

[38] Song Y, Zheng S, Wang J, et al. Hypoxia-induced PLOD2 promotes proliferation, migration and invasion via PI3K/Akt signaling in glioma. Oncotarget. 2017;8(26):41947–41962. 28410212 10.18632/oncotarget.16710 PMC5522040

[39] Brightling CE, Bradding P, Symon FA, et al. Mast-cell infiltration of airway smooth muscle in asthma. N Engl J Med. 2002;346(22):1699–1705. 12037149 10.1056/NEJMoa012705

[40] Berger P, Girodet P-O, Begueret H, et al. Tryptase-stimulated human airway smooth muscle cells induce cytokine synthesis and mast cell chemotaxis. Faseb J. 2003;17(14):2139–2141. 14500550 10.1096/fj.03-0041fje

[41] El-Shazly A, Berger P, Girodet P-O, et al. Fraktalkine produced by airway smooth muscle cells contributes to mast cell recruitment in asthma. J Immunol. 2006;176(3):1860–1868. 16424217 10.4049/jimmunol.176.3.1860

[42] Amin K, Janson C, Boman G, et al. The extracellular deposition of mast cell products is increased in hypertrophic airways smooth muscles in allergic asthma but not in nonallergic asthma. Allergy. 2005;60(10):1241–1247. 16134989 10.1111/j.1398-9995.2005.00823.x

[43] Zhang G, Shao J, Su C, et al. Distribution change of mast cells in human nasal polyps. Anat Rec. 2012;295(5):758–763. 10.1002/ar.22430 22344830

[44] Sheethal HS, Uma K, Rao K, et al. A quantitative analysis of mast cells in inflammatory periapical and gingival lesions. J Contemp Dent Pract. 2014;15(3):300–305. 25307810 10.5005/jp-journals-10024-1532

[45] Banat G-A, Tretyn A, Pullamsetti SS, et al. Immune and inflammatory cell composition of human lung cancer stroma. PLoS One. 2015;10(9):e0139073. 26413839 10.1371/journal.pone.0139073 PMC4587668

[46] Turner CR, Kolbe J, Spannhake EW. Rapid increase in mast cell numbers in canine central and peripheral airways. J Appl Physiol (1985). 1988;65(1):445–451. 3403488 10.1152/jappl.1988.65.1.445

[47] Bull H, Murray PG, Thomas D, et al. Acid phosphatases. J Clin Pathol: Mol Pathol. 2002;55:65–72. 10.1136/mp.55.2.65 PMC1187150 11950951

[48] Vandesompele J, De Preter K, Pattyn F, et al. Accurate normalization of real-time quantitative RT-PCR data by geometric averaging of multiple internal control genes. Genome Biol. 2002;3(7):RESEARCH0034. 12184808 10.1186/gb-2002-3-7-research0034 PMC126239

[49] Delcroix GJ, Garbayo E, Sindji L, et al. The therapeutic potential of human multipotent mesenchymal stromal cells combined with pharmacologically active microcarriers transplanted in hemi-parkinsonian rats. Biomaterials. 2011;32(6):1560–1573. 21074844 10.1016/j.biomaterials.2010.10.041

[50] Monteiro P, Gilot D, Le Ferrec E, et al. AhR- and c-maf-dependent induction of beta7-integrin expression in human macrophages in response to environmental polycyclic aromatic hydrocarbons. Biochem Biophys Res Commun. 2007;358(2):442–448. 17490615 10.1016/j.bbrc.2007.04.111

[51] Lavenus S, Berreur M, Trichet V, et al. Adhesion and osteogenic differentiation of human mesenchymal stem cells on titanium nanopores. Eur Cell Mater. 2011;22:84–96. 21870339 10.22203/ecm.v022a07

[52] Darzi L, Boshtam M, Shariati L, et al. The silencing effect of miR-30a on ITGA4 gene expression in vitro: an approach for gene therapy. Res Pharm Sci. 2017;12(6):456–464. 29204174 10.4103/1735-5362.217426 PMC5691572

[53] Peters C, Häsler R, Wesch D, et al. Human Vδ2 T cells are a major source of interleukin-9. Proc Natl Acad Sci U S A. 2016;113(44):12520–12525. 27791087 10.1073/pnas.1607136113 PMC5098669

[54] Vangipuram SD, Grever WE, Parker GC, et al. Ethanol increases fetal human neurosphere size and alters adhesion molecule gene expression. Alcohol Clin Exp Res. 2008;32(2):339–347. 18162078 10.1111/j.1530-0277.2007.00568.x

[55] Phillips JL Regulation of ITGA6 and ITGB4 integrin genes by RUNX1 and epigenetic mechanisms [PhD thesis]. University of Tasmania; 2017.

[56] Liu JF, Zhou XK, Chen JH, et al. Up-regulation of PIK3CA promotes metastasis in gastric carcinoma. World J Gastroenterol. 2010;16(39):4986–4991. 20954287 10.3748/wjg.v16.i39.4986 PMC2957609

[57] Wagh V, Mishra P, Thakkar A, et al. Antitumor activity of NPB001-05, an orally active inhibitor of Bcr-Abl tyrosine kinase. Front Biosci (Elite Ed). 2011;3:1349–1364. 21622141 10.2741/e338

[58] Karbownik MS, Szemraj J, Wieteska Ł, et al. Antipsychotic drugs differentially affect mRNA expression of genes encoding the neuregulin 1-downstream ErbB4-PI3K pathway. Pharmacology. 2016;98(1–2):4–12. 26960157 10.1159/000444534

[59] Yan LX, Liu YH, Xiang JW, et al. PIK3R1 targeting by miR-21 suppresses tumor cell migration and invasion by reducing PI3K/AKT signaling and reversing EMT, and predicts clinical outcome of breast cancer. Int J Oncol. 2016;48(2):471–484. 26676464 10.3892/ijo.2015.3287 PMC4725461

[60] de Alcântara BBR, Cruz FM, Fonseca FLA, et al. Chemotherapy-induced fatigue is associated with changes in gene expression in the peripheral blood mononuclear cell fraction of patients with locoregional breast cancer. Support Care Cancer. 2019;27(7):2479–2486. 30382394 10.1007/s00520-018-4519-0

[61] McCarthy BA, Yancopoulos S, Tipping M, et al. A seven-gene expression panel distinguishing clonal expansions of pre-leukemic and chronic lymphocytic leukemia B cells from normal B lymphocytes. Immunol Res. 2015;63(1–3):90–100. 26318878 10.1007/s12026-015-8688-3

[62] Untergasser A, Cutcutache I, Koressaar T, et al. Primer3–new capabilities and interfaces. Nucleic Acids Res. 2012;40(15):e115. 22730293 10.1093/nar/gks596 PMC3424584

[63] Ye J, Coulouris G, Zaretskaya I, et al. Primer-BLAST: a tool to design target-specific primers for polymerase chain reaction. BMC Bioinformatics. 2012;13(1):134. 22708584 10.1186/1471-2105-13-134 PMC3412702

